# PROTOCOL: Value chain interventions for improving women's economic empowerment: A mixed‐method systematic review

**DOI:** 10.1002/cl2.1331

**Published:** 2023-06-22

**Authors:** Suchi Kapoor Malhotra, Ashrita Saran, Sabina Singh, Swati Mantri, Neha Gupta, Ratika Bhandari, Howard White, Ranjitha Puskur, Sarah Young, Hugh Waddington, Edoardo Masset

**Affiliations:** ^1^ Campbell South Asia Delhi India; ^2^ Campbell Collaboration, Global Development Network Lanzhou University, Campbell South Asia Vasant Kunj India; ^3^ International Rice Research Institute Manila Philippines; ^4^ Carnegie Mellon University, Research and Academic Services Pittsburgh USA; ^5^ London School of Hygiene and Tropical Medicine London UK; ^6^ Centre of Excellence in Development Impact and Learning (CEDIL) London School of Hygiene and Tropical Medicine London UK

## Abstract

This is the protocol for a Campbell systematic review. The objectives are as follows: The primary objective of this review is to understand as well as evaluate what approaches, strategies or interventions focused on women's engagement in agricultural value chains and markets that have led to women's economic empowerment in low‐and‐middle‐income countries. The secondary objective of this review is to examine in which contexts are these approaches effective (or ineffective)? What are the contextual barriers and facilitators, determining the participation of women in, and benefits from, engagement in the value chain in low‐and middle‐income countries programme effectiveness. Finally, this review aims to refine the theory of change that describes how value chain interventions lead to women's economic empowerment using evidence drawn from both rigorous quantitative impact evaluation studies and qualitative studies.

## 1 BACKGROUND

### 1.1 The problem, condition, or issue

#### 1.1.1 Issue

Agricultural value chains are influenced by socio‐cultural norms and gender dynamics that have an impact on the distribution of resources, benefits, and access to opportunities (Rubin et al., 2010). While women play a critical role in agriculture their place is generally confined to the parts with the least returns of the value chain, or parts of the value chain with the lowest economic returns, as per the social and institutional contexts.

Several development initiatives (both governmental and bilateral) and Results for development programmes have prioritised investment in attaining women's economic empowerment (WEE); they accorded WEE goals via women's participation in agricultural value chains or food systems (as entrepreneurs) and through stronger market engagements. The UN secretary general's panel on women's economic empowerment emphasises the importance of “supporting and enabling women to reach their full potential at all levels of the value chain” (Pyburn & van Eerdewijk, 2021).

There are multiple definitions of economic empowerment. One of the most accepted definitions was advanced by Eyben and colleagues (2008) as ‘the capacity of poor women and men to participate in, contribute to and benefit from growth processes in ways that recognize the value of their contribution, respect their dignity and make it possible to negotiate a fairer distribution of the benefits of growth’. Another definition of relevance is by Golla and colleagues (2011) who emphasize that ‘a woman is economically empowered when she has both the ability to succeed and advance economically and the power to make and act on economic decisions’. Research by Kabeer and Natali (2013) found that though women's entry into the labour market contributes significantly to productivity gains and economic growth. However, the outcomes of economic growth do not essentially result in gender equality or lead to recognition of the power relations construct. Gender dynamics with in value chains operate from individual interactions at the household level up to the level of the value chain, and that of participation‐related issues versus factors that govern levels of gains from participation.

Women have restricted access to resources and information, less control over assets, and heavier workloads, thereby constraining their capacity to engage with and operate within the higher nodes of the value chain (i.e., processing and trading, which often require a minimum number of resources and training) (Forsythe et al., 2016; Meinzen‐Dick et al., 2011). Agricultural value chain interventions aim to create equitable participation in agriclutural markets among men and women by enabling women to overcome the above‐mentioned barriers and facilitating their participation in markets. These efforts support women's economic advancement through more opportunities to realize their entrepreneurial aspirations.

### 1.2 Description of the condition

Traditionally, agricultural interventions have tried to improve farmers’ incomes by increasing farm productivity. This has been pursued, for example, through the introduction of new farming technologies, agricultural extension, and by providing access to inputs such as fertiliser, seeds and loans. However, developments in global market integration over the last 30–40 years suggest that farmers will have a chance to access better income opportunities by strengthening their position within agricultural product markets. For example, farmers may gain considerably by supplying their goods to a large retailer located in a high‐income country, or they may obtain better prices after storing and processing their produce.

The “value chain” is a conceptual framework for understanding agricultural product markets and identifying unexploited non‐farm opportunities. Although the concept of value chain is multivariate in how it is used, for example, by different UN agencies (Stamm et al., 2011); it is primarily used to represent a set of primary and support activities undertaken by an organization to create value for its customers and, thus, to enhance the organization's competitiveness (Porter, 1985). To this end, Kaplinsky and Morris (2000) explained a value chain as 'the full range of activities which are required to bring a product or service from conception, through the different phases of production (involving a combination of physical transformation and the input of various producer services), delivery to final consumers, and final disposal after use.” The simplest characterisation of a value chain, therefore, includes four links: design, production, marketing, and consumption.

**Figure 1:**
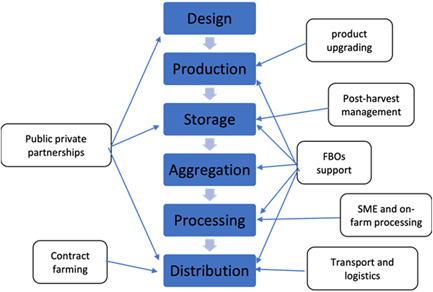
Agricultural value chain and associated interventions. FBOs, farmers' based organizations; SME, small and medium sized enterprise.

However, agricultural value chains are more complex than this simple characterization. For example, the FAO guidelines on “developing sustainable food value chains” include two additional stages between production and marketing: ‘aggregation’ and ‘processing’ (Neven, 2014). Aggregation refers to the process of aggregating and storing the produce of a multiplicity of small farmers by intermediaries, while processing refers to the physical processing of agricultural goods (e.g., pulping and drying coffee beans). Figure 1 illustrates the typical nodes of an agricultural value chain, as well as a tentative classification of ‘value chain interventions’ indicating the node of the chain in which they primarily operate. The tangle of arrows leading from interventions to the nodes in the chain shows how interventions can address constraints at different points in a value chain simultaneously. Value chains are embedded within institutional, economic, social, and natural environments that condition their operation. For example, the movement of products along the value chain will be conditioned by the existing legal frameworks on quality standards, availability of transport infrastructure, or vulnerability to droughts.

Value chains may develop with different levels of complexity. A useful distinction is made between traditional, transitional, and complex value chains (Barrett et al., 2022; De Brauw & Bulte, 2021). Traditional value chains, often related to subsistence crops, are localized and include few nodes, sometimes directly connecting producers to consumers. Transitional value chains, often involving ‘cash crops’, include more nodes in the chain such as storing and processing, and a complicated distribution system. Complex value chains, normally based on internationally traded crops, are articulated in several nodes and include the participation of several actors, some of which are large firms. In complex value chains, goods travel long distances, and relationships between actors are regulated by contracts and other institutional arrangements that certify the quality of the product or insure their value.

**Figure 2:**
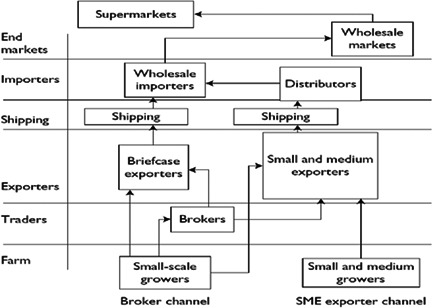
An example of a value chain: A green bean value chain in Kenya. *Source*: Webber & Labaste (2010).

In practice, value chains are much more complex. For example, Figure 2 provides a representation of a value chain of green beans in Kenya. It displays a higher degree of complexity than Figure 1; however, value chain diagrams in the literature tend to be more complex than this. Typically, they are composed of several vertical supply chains which illustrate the journey of a product from conception to consumption. The same product is often supplied through different channels in the same country, resulting in several vertical chains in the same value chain. The functions performed at each link of the chain can be read horizontally: for example, in Figure 2, importers can be ‘wholesale importers’ or ‘distributors’, and end markets may consist of ‘supermarkets’ or ‘wholesale markets.’ The entire value chain is therefore a matrix of functions and channels.

Value chains are built around specific agricultural products. There is no common definition of agricultural products. The FAO FAOSTAT database includes the following items: primary crops (e.g., rice, wheat), processed crops (e.g., cotton, oil, wine), live animals (e.g., goats, pigs), primary livestock products (e.g., milk, meat, eggs), and processed livestock (e.g., butter, cheese, silk). Forestry products include fuel wood, industrial wood and processed woods (e.g., paper, pulp). Fisheries products include all fish varieties, aquatic plants, aquatic animal products (e.g., pearls, corals), aquatic animals (e.g., frogs, crocodiles, crustaceous, molluscs), and mammals (e.g., whales, seals).

Webber and Labaste (2010) and Haggblade and colleagues (2012) provided several examples of value chains including sorghum beer in Botswana, dairy products in Kenya, domestic catfish in Kenya, floriculture in Uganda, green beans in Kenya, cashew in Mozambique, pineapples in Ghana, coffee in Rwanda, cotton in Zambia and avocados in Kenya.

**Figure 3:**
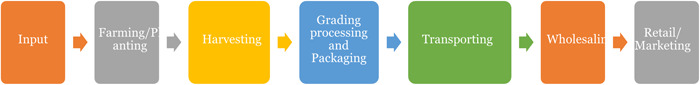
Value chain processes.

The core stage along the value chain are **production**, **aggregation**, **processing**, and **distribution** (wholesale and retail). Embedded in each of the stages are several value chain interventions. As Figure 3, we have mentioned the stages of the value chain all products went through (Morioka & Nicholas, 2014):

In value chain analysis, the following strategies are identified to improve farmers' incomes: vertical coordination, horizontal coordination and upgrading. These can be interpreted as fundamental mechanisms that are expected to operate in isolation or in conjunction with other value chain interventions. All these mechanisms add value to products along the chain and/or allow the actors to capture a higher fraction of the added value. One finds the mechanisms articulated by different terminologies. Multiple terms used to identify the same mechanism (e.g., ‘functional upgrading’, ‘vertical integration’, or ‘value chain deepening’) could affect the clarity with which such discussions occur. Despite the multiple differences in terminologies, we identify the following mechanisms as vital to operation within value chain interventions.

- **Vertical coordination**—This refers to establishing links between market agents along the value chain running from production to final consumption. New links can be established between farmers and buyers that can offer more remunerative prices (contract production). For example, farmers may sign profitable supply contracts with supermarket chains. Further, farmers can also undertake operations related to connected links (vertical integration). For example, producers may take charge of the first stages of processing of the product before selling it, thus adding considerable value to the end product.
- **Horizontal coordination**—Farmers at one particular node of the value chain may aggregate and coordinate activities through farmers’ based organizations (FBOs) or cooperatives. There are many potential benefits arising from coordination, such as an increase in bargaining power when negotiating prices, the exploitation of scale economies (e.g., through collective purchase of machinery), risk pooling, and other reductions in transaction costs.
- **Upgrading**—Activities along the chain can be improved in several ways and authors often distinguish ‘product’ from ‘process’ and ‘functional’ upgrading'. Specifically, farmers may obtain higher prices for their goods by improving quality standards (e.g., through certification, fair‐trade, or organic farming) or producing new goods altogether.^1^ Several efficiency gains can be achieved by organising the production process in different ways or by differently mixing production and processing activities.

The distinction between these types of strategies is not always implicit and overlaps are possible. For example, horizontal integration (the organization of FBOs) may lead to vertical integration (producers’ FBOs taking charge of processing activities along the chain). Product upgrading (e.g., organic farming) may lead to vertical contract production as farmers gain access to specialised buyers. In general, a strategy targeting a particular link in the chain will have ripple effects on other links, and value chain interventions will often encompass a combination of the strategies above.

More recently, strategies for gender‐sensitive and gender‐responsive value chain interventions have been developed by several agencies and international NGOs. These Table strategies, as discussed below, aim to provide consolidated support for women to access the benefits offered by value chain interventions.

First, the gendered nature of tasks in rural economies often push women toward less remunerative nodes in the value chain, such as agricultural labor, petty trading, and subsistence farming. There is scope to strengthen the value chain positions currently occupied by women and to enable their participation in more remunerative positions. However, in order to do this, it is first essential to identify and address the barriers faced by women when willing to participating in the agricultural value chain.

Second, women are less likely to adopt new technology in their activities primarily because of the very reasons that prevents their participation in profitable nodes in the value chain.

To this end, the FAO guidelines for sustainable value chains in particular list the following factors: poor access to information and training, limited participation and decision‐making power within household and communities, limited access to financial services and other productive assets and resources, and excessive work burden. These factors are in turn embedded in reinforcing cultural norms and institutions. Hence, interventions aiming to make the services accessible for women is required.

We can identify three principal ways in which agencies can promote gender‐responsive value chain interventions. By gender‐responsive we mean interventions that in their design and implementation employ gender consideration (United Nations Children's Fund, 2017). This definition will include gender‐transformative interventions directly addressing structural barriers and power relations (Cole et al., 2020) as well as more gender‐accommodative interventions that recognize gender constraints and that try to release some of these constraints but without addressing structural barriers (Interagency Gender Working Group, 2017).

- **Promote women‐led nodes in value chains**. Rural economies usually operate via gender‐ specific roles. Analysis of any value chain suggests that some links are entirely occupied by male farmers, while others are dominated by women. For example, women may oversee the processing a staple crop, or retailing. An intervention may therefore decide to strengthen the node in the chain which is dominated by women to achieve the maximum benefit for them.
- **Promote women's participation in male‐dominated value chains**. An intervention may decide to promote a particularly remunerative node of the value chain, for example linking FBOs to buyers connected to export markets. However, this link may be dominated by male farmers. The intervention can therefore promote women's participation in FBOs and support their presence in leadership roles, in a way that shares the benefits of the intervention across gender groups. The intervention may attempt to specifically promote the inclusion of women in highly profitable value chains or more profitable nodes of the value chain that are traditionally controlled by men.
- **Address discriminatory constraints to access to value chains**. Selecting value chain activities that are dominated by women and promoting their participation in the project activities may be insufficient. Fundamental constraints must be identified that prevent women from participating or enjoying the benefits of project activities. The value chain intervention may include specific complementary interventions that are oriented toward increasing women participation in markets and in decision‐making capacities. For example, a project might include loans specifically targeted at women, or make provisions for childcare to enable effective participation of women in the activities.

The gender‐responsive value chain intervention that we will consider in our review are:

- Value chain interventions explicitly targeting women;
- Interventions that promote and **support women's participation in value chain domains that are traditionally male dominated; and**
- Interventions that **address constraints that prevent women from participating** or accessing benefits.

### 1.3 Description of the intervention

The review will include interventions that engage women in agriculture value chain processes in different capacities‐ whether by creating an enabling environment to engage women farmers or focusing on land access and ownership, changes in policies, and social norms (Doss & SOFA Team, 2011).

A review of value chain strategies by UN agencies (Stamm et al., 2011) and international NGOs (Webber & Labaste, 2010) led to identification of the ‘value chain’ interventions in Table 1 (classified by the main strategy above). The goal of value chain intervention is to improve farmers’ incomes, particularly those of small farmers. This could be achieved through factors such as better integration into markets, access to more remunerative prices, development of new and more profitable products, and adoption of functions adding value to production, such as processing or storage. The review will focus on analysis of value chain interventions that are also gender‐responsive.

**Table 1:** List of interventions.

Value chain strategy	Interventions
Vertical coordination	Contract farming Public‐private partnerships
Horizontal coordination	Promotion of FBOs and Coops
Upgrading/and others	Post‐harvest management (SME) processing development Product quality upgrading (e.g., certification, organic, fair trade) Inclusive market systems development Infrastructure development (e.g., transport) Storage and On‐Farm Processing

Abbreviations: FBO, farmers’ based organization; SME, Small and medium sized enterprise.

### 1.4 How the intervention might work

In this review, we have adopted the causal chain analysis, in which we will analyse the theory of change for an intervention. We will also synthesise the findings of process evaluations and qualitative studies to identify both the barriers and facilitators to project implementation.

We have built the theory of change (ToC), also referred to as middle‐level theory (MLT). It is appropriate for systematic reviews that look at different settings and populations to identify and test the assumptions under which specific interventions generate the outcomes (White, 2018).

The analysis approach is based on specifying the logic model for intervention. It will also address the following questions:

- How does the intervention work (mechanism or methodology of intervention)?
- Why the intervention works (causal pathway)?
- What are the weakest and/or missing links in the causal chain/pathway?

The theory of change is to enable women's economic empowerment through value‐chain interventions. This could be achieved by providing access to resources, an enabling environment, agricultural inputs, and capacity building. Here, an attempt has been made to explain the causal pathway of how the engagement of women in all stages of the value chain (from production to distribution) may result in their economic empowerment.

Gender empowerment is defined as ‘a process by which those who have been denied the ability to make strategic life choices acquire the ability to do so’ (Senders et al., 2012). Although the methods and domains used to measure women's empowerment may vary across studies and are context specific, the component of agency is of crucial significance. Therefore, in any value chain process, the intervention should consider the various dimensions of the concept. This would not only enable impact assessments of the intervention but would acknowledge the dynamism of the context in which the intervention is introduced. To take this discussion further, let us briefly outline the important dimensions of the concept.

First, women's empowerment is understood as a process, and not an end in and of itself. It refers to women's ability to choose three interrelated elements: resources, agency, and achievement. Resources such as money, education, social capital, land, and credit strengthen the empowerment process by enabling women's agency. Agency refers to the ability to formulate strategic choices and control over resources and decisions. It has been believed that the precondition to achieving gender equality lies in women's agency and decision‐making power (Okesina, 2020; Senders et al., 2012).

The second aspect of women's empowerment is that of power relations. There are four types of power relations: ‘power over’, ‘power within’, ‘power to’, and ‘power with’ (Okesina, 2020; Senders et al., 2012). ‘Power over’ refers to a zero‐sum situation (i.e., one person's gain is another person's loss), which is best explained in patriarchal societies in which men control resources and decision–making. While ‘power within’ refers to a state of confidence, dignity and self‐esteem. ‘Power to’ refers to the capacity to make desired change‐ the ability to exercise choice and change one's condition. ‘Power with’ refers to networks, groups and collective strength‐based collaboration and solidarity, and could be understood as a win‐win situation for both genders (Okesina, 2020; Senders et al., 2012).

Women's empowerment in terms of a power approach suggests that a combination of ‘power within’, ‘power to’, and ‘power with’ is necessary to arrive at gender equity. Such dynamics suggest that one's agency to make effective choices subtly hints at a change in power relations as well as in that individual's autonomy and decision‐making capacity. ActionAid employs a similar concept to gauge women's empowerment. It is referred to as ‘Power cube 5’, and analyses power constructs via the indices of ‘power within’, ‘power with’, and ‘power to’ (Senders et al., 2012; Morioka & Nicholas, 2014; Powercube, 2013).

The third aspect of women's empowerment is the that of an outcome. Empowerment means that there are definite outcomes in terms of change in economic, social, and political constructs‐ not only in a change in processes or in power relations. Achievement aims to increase labor participation and educational attainment (Senders et al., 2012).

The fourth aspect of women's empowerment is a multi‐dimensional concept. It comes into effect from different dimensions such as personal, relational, micro, meso, and macro levels (Okesina, 2020; Senders et al., 2012).

Oxfam defined that effective ‘women's economic empowerment can be achieved when women enjoy their right to control and benefit from resources, assets, income and their own time, and when they can manage risk and improve their economic status and wellbeing’ (Kidder et al., 2017). This is exemplified when women also have autonomy and self–belief, as well as agency and power to influence decision‐making and make change in their lives. To understand gender in value chains, we must bring together a value‐chain development approach and gender right‐based approach together. Below we explain the Chain empowerment matrix through a gender lens (Senders et al., 2014).

#### 1.4.1 Chain empowerment matrix

The chain empowerment matrix s a useful framework to explain chain development (KIT, Faida MaLi, & IIRR, 2006). There are two broad dimensions to understand the types of participation within a chain. While chain activities refers to those undertaken by farmers (i.e., who does what); chain governance refers to farmers' involvement in chain management (i.e., who determines how things are done) (Senders et al., 2012).

**Figure 4:**
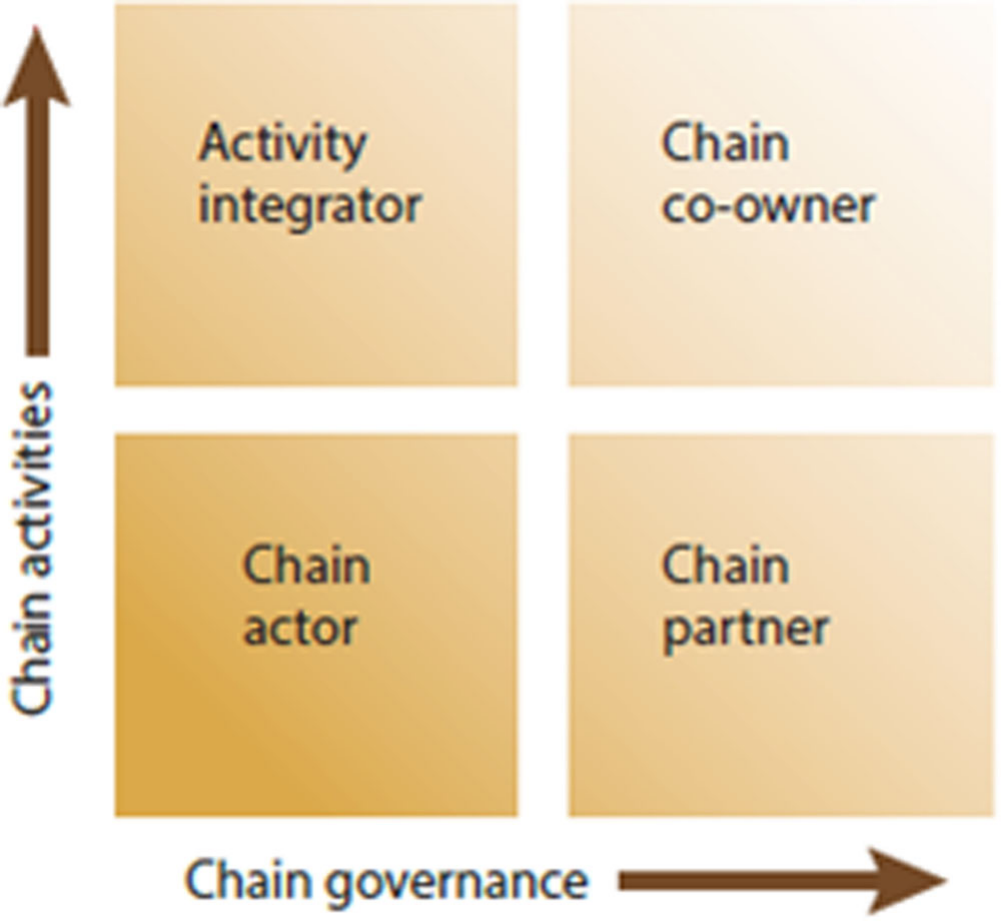
Chain empowerment matrix. *Source*: KIT Faida MaLi & IIRR,  2006.

To explain further, farmers can undertake different activities such as drying, fermentation of their crops (post‐harvest activities) or grading, processing, transporting, and trading. Vertical integration/coordination is understood as involvement in various activities in the chain. The farmers concerned with management of the chain are usually related to the tasks of decision‐making processes and control over management issues. Farmers are in control to decisions such as sale quantities and prices. Involvement in multiple chain management issues is known as horizontal integration/coordination (Senders et al., 2012) (Figure 4).

Participants ability to conduct value chain activities (e.g., processing, post‐ harvesting) more efficiently and profitably could result in chain empowerment. It reduces transaction costs by integrating activities that were previously handled or controlled by others. They gain control over the value chain processes through improved negotiation capacities and build stronger relationships with other stakeholders in the value chain (Morioka & Nicholas, 2014).

Four dimensions of gender empowerment in value chains are mentioned below. For this review, we focus on economic empowerment (Table 2).

**Table 2:** Dimensions of gender empowerment.

Vertical integration into chain	What activities do women and men in the chain do?
	What benefits do women and men gain?
Horizontal integration into chain	Who determines the conditions under which these activities are performed, and How benefits are gained and distributed?
Gender dynamics in household and community	How do changes in the first two dimensions affect the gender division of labor, assets, and decision–making within the household?
	How do the changes in the first two dimensions affect the gender dynamics within the community?
Individual context: rules, norms, and values	Which economic, political, and social factors enable or constrain women's empowerment in the other three dimensions?
	How do changes in the first two dimensions influence the institutional context?

It is important to note that globally, approximately 75% of women farmers play active roles as producers, traders, processors, laborers, and entrepreneur; however, they are disadvantaged as their roles are largely unrecognized (Gurung et al., 2015). This is due to the many constraints they face such as limited access to land, irrigation, productive inputs, financial credit, property rights, new technologies and market (Agarwal, 2018; Doss & SOFA Team, 2011; Gurung et al., 2015; Mayoux & Mackie, 2008; Mutua et al., 2014; Rubin et al., 2010; Ugwu, 2019).

The evidence suggests that gender differences and inequalities exist at all levels of the value chain, and act as the main barrier to women's participation in agricultural activities (Ugwu, 2019) (Gurung et al., 2015). Other barriers include: (1) customs, beliefs, and attitudes that confined women to the domestic sphere; (2) women's workload and time poverty; and (3) laws that affect access to resources, employment, and education. It has been further elaborated that women's participation in markets is restricted due to a lack of accessible transportation, as well as social norms prohibiting them from traveling (Mayoux & Mackie, 2008; Quisumbing et al., 2013; Ugwu 2019). The intended outcomes of some value chain interventions entail addressing of these constraints‐ particularly regarding increasing women's free time, mobility, income and decision making.

For the theory of change, we combine the existing approaches and focus on interrelated dimensions, access to productive resources, power, and agency. The same approach was adopted by FAO (2016). The visual representation shows the causal process through inputs, meaning access to productive resources which facilitate activity implementation, (value chain interventions in horizontal and vertical coordination and upgradation), which in turns help to achieve the immediate outputs as a Chain actor (contributions are recognised and valued) activity Integrator (increase in knowldge and skills and control over earned income), Participation in decision making power (chain partners) and this finally helped to achieve the impact (Women's economic empowerment).

The left side of the figures lists the value chain interventions:

- Vertical coordination (contract farming production);
- Horizontal Coordination (promotion of farmer‐based rganizations (FBOs)); and
- Upgrading (fair trade, upgrading product quality).

The objective of the value chain intervention mentioned are as follows:

1. To enhance women's roles in agricultural value chains (e.g., increasing their involvement in specific nodes or stages of the value chain such as processing or marketing); and
2. To expand opportunities for women (Quisumbing et al., 2013).

**Figure 5:**
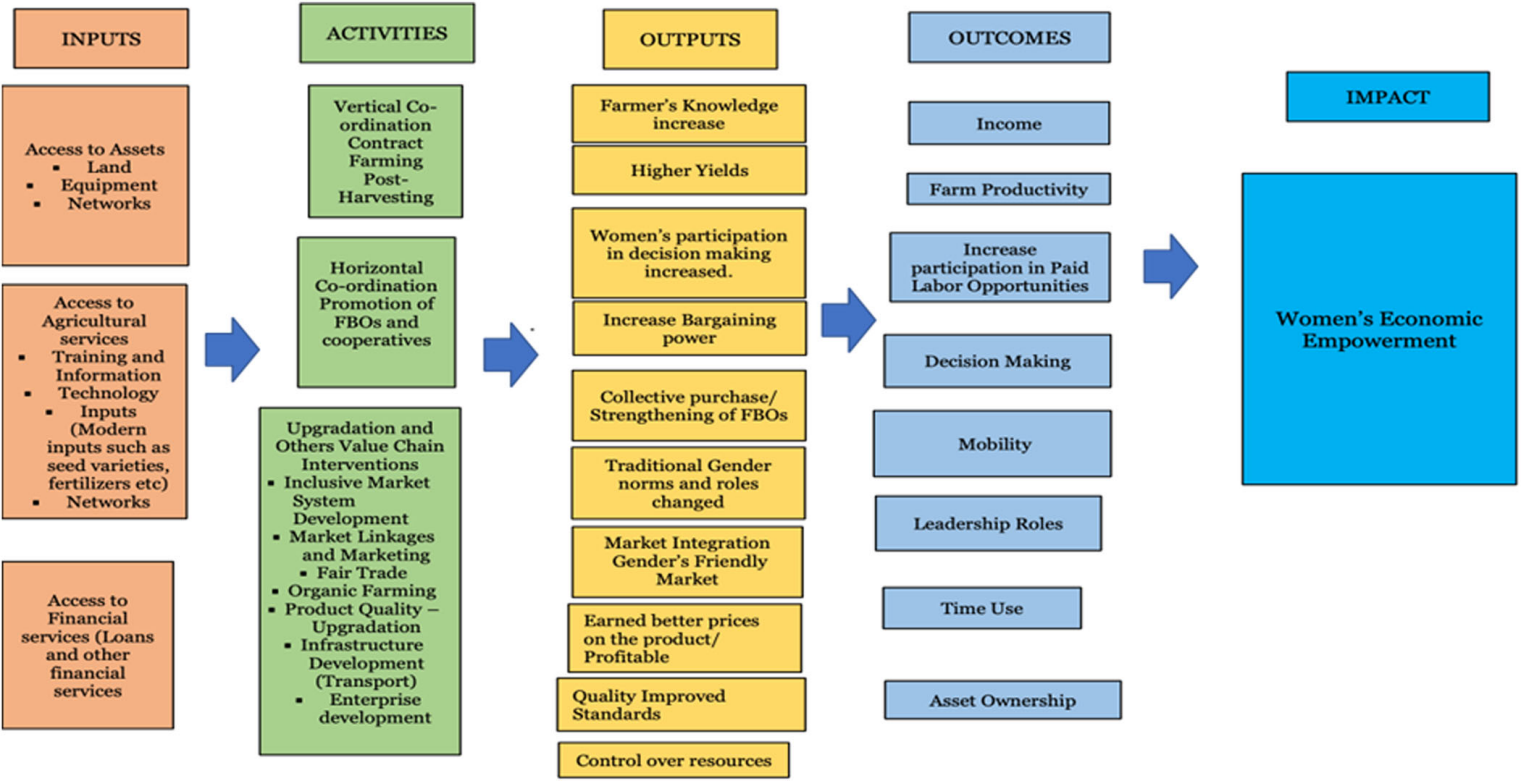
Theory of change.

This theory of change explains the inputs and activities required to attain the desired intermediate outcomes and, ultimately, women's economic empowerment. The casual pathways in Figure 5 can be followed from left to right. In the proposed theory of change, we illustrated that access to inputs (eg., fertilizers, credit, loans, access to information, and training) can lead to the intended immediate outputs. The value chain interventions may provide or promote women's contributions and aim for their economic empowerment (e.g.,, processing or storage technologies that add value to the product, which we would consider this under 'post‐harvest management' and ‘processing development’ interventions). The approach of ‘power with’ ‐ such as the establishment and strengthening of the farmer's group, collectives, and more female farmers’ groups‐ helps to improve income, decision‐making, bargaining, women's participation, and economic empowerment.

Embedded in each of the stages are several value chain interventions; our theory of change explains these interventions and how they may lead to women's economic empowerment. We assume that women's participation in the value chain as actors, partners, and co‐owner offers opportunities for economic benefits and empowerment.^2^

There are various stages of the value chain processes and involving them all in this ToC involved the risk of being extremely complicated. In the protocol, we listed classifications of interventions and intermediate outcomes. The current TOC is tentative, and one aim of our work is to build one that is theory‐informed (or middle‐range).

To achieve the outcomes‐ while considering empowerment as an outcom‐, we have some assumptions. The first is that women have access to resources such as irrigation, productive inputs, financial services, and new technologies. It has been also assumed that women actively participate and have access to knowledge and training. There is evidence that women engaged in farming are often exploited by other actors in the value chain (buyers and suppliers) due to their high level of illiteracy, weak bargaining power, lack of knowledge, and access to market information (Gurung et al., 2015). We assumed that the value chain interventions helped to address the above‐mentioned constraints to achieve the intended outcomes (Figure 5).

### 1.5 Why it is important to do this review

A literature review suggests that there are no systematic reviews on this topic. Several reviews address similar or overlapping questions including the following:

Gibbs, A., Willan, S., Misselhorn, A., & Mangoma, J. (2012). Combined structural interventions for gender equality and livelihood security: A critical review of the evidence from southern and eastern Africa and the implications for young people. *Journal of the International AIDS Society*, *15*, 1–10.

Riisgaard, L., Fibla, A. M., & Ponte, S. (2010). Evaluation study: gender and value chain development. *Copenhagen: The Evaluation Department of the Danish Foreign Ministry*.

Stewart, R., Van Rooyen, C., Korth, M., Chereni, A., Da Silva, N. R., & De Wet, T. (2012). *Do Micro‐credit, Micro‐savings and Micro‐Leasing Serve as Effective Financial Inclusion Interventions Enabling Poor People, and Especially Women, to Engage in Meaningful Economic Opportunities in Low‐and Middle‐income Countries? A Systematic Review of the Evidence*. EPPI‐Centre.

As is evident, there remains a significant gap in evidence on the impact of women's engagement in agricultural value chains and markets on their economic empowerment and the barriers to achieving this.

## 2 OBJECTIVES

- The primary objective of this review is to understand as well as evaluate the approaches, strategies or interventions focused on women's engagement in agricultural value chains and markets that have led to women's economic empowerment in low‐and‐middle‐income countries.
- The secondary objective of this review is to examine the contexts in which these approaches are effective (or ineffective)? What are the contextual barriers and facilitators determining women's participation in, and benefits from, engagement in the value chain in low‐and middle‐income countries?
- Finally, this review aims to refine the theory of change that describes how value chain interventions lead to women's economic empowerment, using evidence drawn from both rigorous quantitative impact evaluation studies and qualitative studies.

## 3 METHODS

### 3.1 Criteria for considering studies for this review

#### 3.1.1 Types of studies

To answer Objective 1, we will use an experimental or quasi‐experimental design. Eligible designs include those in which the authors use a control or comparison group and in which one of the following is true:

- Participants are randomly assigned (using a process of random allocation, such as a random number generation);
- A quasi‐random method of assignment has been used and pre‐treatment equivalence information is available regarding the nature of the group differences (and groups generated are essentially equivalent);
- Participants are non‐randomly assigned relevant demographic characteristics (using observables, or propensity scores) and/or according to a cut‐off on an ordinal or continuous variable (regression discontinuity design); or
- Participants are non‐randomly assigned, but statistical methods have been used to control for differences between groups (e.g., using multiple regression analysis, including difference‐in‐difference, cross‐sectional (single differences), or instrumental variables regression).

No restriction will be placed on the duration of follow‐up.

The evidence incorporated under Objective 2 will be broader to include that present in impact evaluations, as well as studies collecting and analyzing qualitative evidence. Answering Objective 3 will involve the examination of plausible pathways, barriers, and facilitators of included studies across contexts from the evidence included in the review, and a conceptual framework will be articulated.

#### 3.1.2 Types of participants

Participants included women of all ages from low‐ and middle‐income countries engaged in agriculture and food systems and targeted by value chain development and market engagement interventions.

Population sub‐groups included smallholder farmers, value chain entrepreneurs, or employees in the agribusiness sector.

Low‐ and middle‐income countries were identified according to World Bank categorization at the time the data were collected.

#### 3.1.3 Types of interventions

The interventions of interest under this review are those aiming to empower women through value chain development and market engagement implemented in agriculture. These interventions aim at increasing women's market participation in the value chain by removing barriers to participation, or by changing social norms in organizations, communities, and families in order to facilitate their participation.

The interventions may include the following:

- Inclusive market systems development;
- Horizontal coordination (Promotion of FBOs and Coops);
- Vertical coordination (Contract farming, Public‐private partnerships);
- Post‐ harvest management;
- Gender‐friendly markets (lighting, washroom facilities, provision for childcare);
- Enabling policies and institutional environment;
- Financial services (both grants and subsidies, and micro‐credit, savings and insurance);
- processing and storage facilities;
- Process, product, and chain upgrading;
- Enterprise development and impact investing;
- Infrastructure development (transport);
- Promoting the production of a new profitable product;
- Improving product market quality (fairtrade, organic farming, quality standards);
- Supporting horizontal integration of producers' groups to access better prices; and/or
- Improving processing techniques (on‐farm processing).

#### 3.1.4 Types of outcome measures

The outcomes of interest for this review include measures that result in women's economic empowerment and the various dimensions embedded in that. The listed indicators reflect the main areas of empowerment within agricultural value chains and markets.

Not all studies will report impact in terms of empowerment outcomes. We will also consider other outcomes along the project causal chain. These do not characterise empowerment but are often preconditions.

##### Primary outcomes

- Economic empowerment outcomes, such as decision‐making on value chain activities, decision‐making over the use of income, leadership positions in groups and increased bargaining power; and
- Economic outcomes, such as incomes, prices and market sales.

##### Secondary outcomes

Eligible secondary outcomes include participation‐related outcomes mentioned in Table 3.

**Table 3:** Outcomes categories and sub‐categories.

Outcomes	Indicators
Economic Empowerment	Multidimensional empowerment indices such as the women's empowerment in agriculture index (WEIA) and other ad‐hoc indices built by researchers
	Agency indicators: Decision‐making on value chain activities, decision‐making over the use of income, increased bargaining power;
	Leadership positions in groups;
	Mobility
Economic benefits	Farm productivity
	Income
	Time use
	Assets ownership
Participation	Access to information on production/markets
	Enhanced social and institutional networks
	Increased participation in paid labor opportunities
	Access to new markets
	Knowledge and skills
	Gender roles and norms

### 3.2 Search methods for identification of studies

The electronic searches of the selected databases and gray literature search using organisational websites. And hand searches of selected journals and articles will also be done.

#### 3.2.1 Electronic searches

We have devised a search string to capture the relevant completed and ongoing studies. We will search the following databases:

Web of Science Core Collection (Social Sciences Citation Index (SSCI), Science Citation Index Expanded (SCI‐EXPANDED), Conference Proceedings Citation Index—Science (CPCI‐S), Conference Proceedings Citation Index—Social Science & Humanities (CPCI‐SSH), Emerging Sources Citation Index (ESCI (Clarivate Platform), Scopus (Elsevier), International Bibliography of the Social Science (ProQuest), EconLit, Business Source Premier, APA PsycInfo (EBSCO), Cochrane Library (Central Register of Controlled Trials, Database of Systematic Reviews), CAB Abstracts (CAB Direct), and Sociological Abstracts (Pro Quest).

In Supporting Information: Appendix A we present the example of the search string used for the Scopus database.

#### 3.2.2 Searching other resources

We will also search websites of the relevant organisations for ongoing and completed studies. We will also identify and search any relevant existing reviews to locate additional primary studies. A full list of these organizations and websites is provided in Supporting Information: Appendix B. We will also conduct a machine learning assisted search in EPPI‐ Reviewer Beta Version (Open Alex). Open Alex dataset, like Google Scholar, is a comprehensive repository of research articles containing 250 million bibliographic records. It searches not just academic databases but also search grey literature sources.

### 3.3 Data collection and analysis

***Description of methods used in primary research***

The systematic screening will be carried out for the search studies based on the screening tool. And systematic data extraction will be done for the final included studies in the review based on the data extraction tool. The details procedure is described below:

#### 3.3.1 Selection of studies

*We will systematically screen all records using a screening tool*. The screening for inclusion/exclusion will be undertaken in two stages using EPPI *R*eviewer 4, *a custom‐built software for screening and coding*. The first stage is the title and abstract screening, and the second is the screening of the full text. The first stage will be assisted by priority screening, which is the machine learning function in EPPI. Both stages will be performed by two independent researchers using the screening tool, with a third‐party arbitrator in case of disagreement. The screening tool is included in Supporting Information: Appendix C.

In this stage, we will identify the eligible records for this review. The screening will allocate studies into one of two groups. (1) Studies reporting the intervention's effects; and (2) Studies reporting barriers and facilitators. We will develop the screening tool for the review using the agreed PICOS for the inclusion and exclusion criteria. The screening tool will be piloted.

#### 3.3.2 Data extraction and management

The standardized data extraction form will be used to extract data from all the included studies (Supporting Information: Appendix D). The form will be piloted and modified as necessary following the ‘revise, refine, and define’ approach described in the Campbell guidance. The form includes geographical information, population, study design and method, intervention type and outcome types, and sub‐categories. The data extraction will be conducted by two researchers, and any disagreements will be resolved through discussion with the third reviewer, as needed.

The data extraction tool for this review also includes raw data for meta‐analysis. The meta‐analysis data extraction will be done in Excel.

#### 3.3.3 Assessment of risk of bias in included studies

The study's confidence will be assessed using a critical appraisal tool for primary studies developed by the Campbell Collaboration Secretariat. The tool can cover both quantitative and qualitative studies (Supporting Information: Appendix E). It was developed by the Campbell Collaboration along with their partners at the Early Intervention Foundation (EIF), and l was created by consulting other quality tools available (namely the Critical Appraisal Skills Programme Checklist [CASP]) and inputs from experts in the field.

Coding for critical appraisal will be performend by two independent reviewers. The tool includes critical dimension of the evaluation, each of which is marked as high, medium, or low. The overall score uses the ‘weakest link in the chain’ principle. Hence, confidence in study findings can only be as high as the lowest rating given to the six critical items in the effectiveness study and the nine critical items in the qualitative/process evaluation.

#### 3.3.4 Measures of treatment effect

We will extract comparable effect size estimates with 95% confidence intervals from the included studies. Effect sizes will be measured as the standardised mean difference (SMD) for continuous outcomes, or the odds ratio (OR) for dichotomous outcomes. Treatment effects will be calculated as the ratio of, or difference between, treated and control observation in a consistent way, such that an increase in the outcome measure reflects a desirable change. Thus, effect sizes where the SMD is greater than zero (an OR greater than 1) will indicate a positive effect of the intervention on the outcome. An SMD less than zero (an OR between 0 and 1) will indicate a negative effect. The positive or negative effects depend on the meaning of the outcome, higher yields or an increase in income is positive effect of the intervention, and an increase in use of pesticide and a lack of access to resources are negative effects. For an undesirable outcome, the SMD is multiplied by −1 or the OR is inverted.

The analyses will be estimated based on the outcome category, and subsequently by outcome subcategory. To generate forest plots at the outcome level, we first calculate the single synthetic effect size by taking the weighted average of effect across subcategories of the outcome.

#### 3.3.5 Unit of analysis issues

The unit of analysis for quantitative data will be individuals or households. Where the study uses clustering of treatment units (e.g., at the village level), we will apply standard unit of analysis error correction formulae to ensure that the standard errors of the SMD or OR are based on the effective sample size (Higgins et al., 2011).

#### 3.3.6 Criteria for determination of independent findings

Multiple papers or reports based on the same study or data will be treated as a single case. The report or the paper will only be treated as a separate case if the study sample does not include study participants included in any other coded study. Where there are multiple papers or reports, we will select the revised or updated version, if all of the relevant information is available in a single source. However, if the multiple reports provide different information (e.g., different outcomes or different subgroups), then the data from all these reports will be coded as a single case, taking different information from each study. To avoid double‐counting of effects from the same study, we will include only one effect estimate per a study in each meta‐analysis, unless the effects are from independent interventions measured against a single control group‐ in which case we will split the control sample in two, assuming the same mean difference or frequency in each control sample.

#### 3.3.7 Dealing with missing data

Study authors will be contacted if we require additional data that is missing or incomplete to calculate the effect size. In case of non‐availability or no response from authors, we will report the characteristics of the study, but will not include such a study in the meta‐analysis.

#### 3.3.8 Assessment of heterogeneity

Relative and absolute heterogeneity between effect sizes will be assessed by reporting I‐ squared (*I* ^2^) and tau‐ squared (*τ* ^2^) statistics. Forest plots will be generated for a visual representation of the individual study effects and the pooled effect size. We will use moderator analysis and meta‐regression to analyze the possible causes of heterogeneity, if any.

#### 3.3.9 Assessment of reporting biases

Publication‐selection bias will be assessed for the primary outcomes by constructing a funnel plot for each outcome (Higgins et al., 2011), which will be presented alongside Egger's regression test lines of best fit.

#### 3.3.10 Data synthesis

In this review, we will compute the SMD from the available information found in primary studies such as means, regression coefficients, or chi‐squared (*χ* ^2^) tests from analysis of variance. We will compute the OR from available information on proportions and frequencies. The effects size calculations will be performed using an effect size coding tool developed for this review, and, in a minority of instances, the Campbell online effect size calculator (Wilson, 2011).

Meta‐analysis of effect sizes for each outcome will be conducted using Stata. A weighted mean effect size for each outcome will be reported under a random‐effects model. Overall effect sizes for primary outcomes will be reported in units of the standard deviation of the outcome to communicate with policymakers and practitioners.

#### 3.3.11 Subgroup analysis and investigation of heterogeneity

Moderator analyses of a single categorical variable will be conducted using subgroup analysis, analogous to an analysis of variance (ANOVA), also under a random‐effects model. Moderator analyses of continuous moderators for multiple moderators will be conducted using meta‐regression analysis and reported under a random‐effects model.

Our a priori planned moderator analyses include: (i) targeted interventions versus non‐ targeted interventions; (ii) types of the research design; and (iii) region. Post hoc moderator analyses may be estimated if the qualitative synthesis suggests patterns of heterogeneity in the data that may be explored in quantitative analysis.

#### 3.3.12 Sensitivity analysis

The sensitivity analysis will be carried out by removing studies from the meta‐analysis one‐by‐one to see if the results of the meta‐analysis are sensitive to any single study. We will also examine the sensitivity of findings by risk of bias (low risk, some concerns and high risk).

#### 3.3.13 Treatment of qualitative research

In this review, we will adopt the approach of combining the qualitative data with the quantitative meta‐analysis, within the framework of a theory‐based systematic review TBSR (White, 2018). In the framework, the intervention is the unit of analysis, rather than the individual study. Different studies may contribute findings at different stages of the casual chain. For example, process evaluations and qualitative studies provide more evidence on implementation issues than most effectiveness studies, such as the failure of establishing women's involvement in the programme and why, which can help explain both the size of and variations in, effect sizes.

The TBSR framework is shown in Table 4. Quantitative data are indicated as Qt and qualitative as Ql. Quantitative data refers to both effect sizes and factual quantitative data such as participation rates.

**Table 4:** Theory‐based systematic framework.

	Participation	Activities	Enabling Environment	Services	Economic Impact and Participation	Women's empowerment
Case 1						Horizontal synthesis
Case 2						
‐‐‐						
Case n						
	Vertical synthesis					Overall synthesis

Table 4 shows the TBSR framework which is used for both horizontal and vertical synthesis (White, 2018). The data in Table 5 are subject to vertical, horizontal, and total synthesis.

**Table 5:** Stages of the causal chain with data to be examined at each stage.

Stage in the causal chain	Data
Awareness of the programme amongst relevant service providers and target group	Know of programme, aware of eligibility criteria, purpose, and how to access (Qt/Ql)
Activities undertaken	Descriptive material (Ql)
Connection to services (access to markets, credit/savings)	Access to services (Qt and QI)
Enabling environment	Change in social norms (Ql)
Strengthen the existing groups/and more involvement of women	Farmer groups, co‐operatives, and women's involvement/engagement (Qt and Ql)
Economic impact and participation	Increase in income, agricultural productivity, ownership, skills, and knowledge. (Qt supported by Ql).
Women empowerment	Women's economic empowerment. (Qt and QI)

Vertical synthesis involves summarizing the evidence across all cases, which is the way in which systematic reviews are usually performed, especially for quantitative analysis of effects. In the case of qualitative data, vertical synthesis is a thematic analysis, in which common themes are identified across studies.

Horizontal synthesis summarizes across a case—which may be done in narrative reviews; however, the difference here is that the data for an intervention may come from more than one study.

The overall synthesis combines both, though it may well contain separate overall synthesis by sub‐group. The overall synthesis approach, drawing on both horizontal and vertical synthesis, ‘tells the story’ of whether the intervention works, for whom, under what circumstances, and why.

## CONTRIBUTIONS OF AUTHORS

### Roles and responsibilities

Content: Ranjitha Puskur, and Edoardo Masset are responsible for content. Edoardo Masset is also the technical lead for the review.

Systematic review methods: Edoardo Masset, Howard White, and Suchi Kapoor Malhotra are responsible for ensuring that satisfactory systematic review methods are used.

Statistical analysis: Hugh Sharma Waddington will lead on statistical analysis. Suchi Kapoor Malhotra and Neha Gupta will assist.

Qualitative data analysis: Suchi Kapoor Malhotra and Swati Mantri are responsible for performing qualitative data analysis.

Information retrieval: Sarah Young, informational retrieval specialist, designed the searches based on the suggestion by Edoardo and Suchi. She is responsible for information retrieval on the databases.

## DECLARATIONS OF INTEREST

Howard White is CEO of the Campbell Collaboration. As CEO he has no role in the editorial process.

## PRELIMINARY TIMEFRAME

- Date you plan to submit a draft protocol: December 2021
- Date you plan to submit a draft review: June 2022

## SOURCES OF SUPPORT

Internal source

New Source of support, Other

No sources of support provided

**External sources**

This study is funded by the CGIAR Generating Evidence and New Directions for Equitable Results (GENDER) Platform form.

## Supporting information

Supporting information.

## Footnotes

^1^
Product upgrading strategies comprise activities meant to improve a product's quality and increase its value for consumers or other intermediary use, whereas process upgrading strategies focus on enhancing production efficiency using improved production technology or better coordination of the production process. Further, functional upgrading strategies allow a value chain actor entry into a new and more enhanced value‐added function in the value chain.

^2^
This notwithstanding, Ihalainen et al. (2021) stated instances where: (1) participation in value chains and value chain development can have disempowering effects; and (2) empowerment and disempowerment can run concurrently and produce contradicting effects within and beyond the value chain as well as between different dimensions of empowerment.
