# Supplementary material for: PROTOCOL: Value chain interventions for improving women's economic empowerment: A mixed‐method systematic review
Source: Campbell Syst Rev. 2023 Jun 22;19(3):e1331. doi: 10.1002/cl2.1331 (PMC10286722; doi:10.1002/cl2.1331)
Supplement: Supplementary file 1 — Supporting information. [file CL2-19-e1331-s001.docx]

Appendices

1 Appendix A- Search terms

Database name: Scopus

Platform: Elsevier

| 1  Agriculture | TITLE-ABS-KEY(“smallhold*“ OR “small hold*“ OR “microfarm*“ OR “micro-farm*“ OR “pastoral*“ OR “agropastoral” OR “agro-pastoral” OR “ejido” OR “silvopastoral” OR “farm*“ OR “agricultur*“ OR “producer*“ OR “grower*“ OR “agronomy” OR “husbandry” OR “aquacultur*“ OR “floricultur*“ OR “horticultur*“ OR “cultivat*“ OR “dairy” OR “livestock” OR “crop*“) | 2,070,995 |
| --- | --- | --- |
| 2  Value-chain | TITLE-ABS-KEY(fairtrade OR “fair trade” OR “contract farming” OR “post-harvest management” OR “making markets work” OR “market system*“ OR “Participatory Market Chain Approach” OR “market development*“ OR “market intervention*“ OR “agricultural product market*“ OR “supply chain*“ OR “production to consumption system*“ OR “farmers’ based organisations” OR “farmers’ based organization*“ OR “farmer based organization*“ OR “farmer based organization” OR “farmers’ group*“ OR “farmers group*“ OR “cooperative*“ OR “value-add*“ OR “value-chain*“ OR “market modernization” OR “market modernisation” OR “modern market*“ OR “ marketing channel*“ OR “commercialization channel*“ OR “commercialisation channel*“ OR “high-value chain*“ OR “high-value market*“ OR “agrifood transformation*“ OR “agri-food transformation*“ OR “agrifood system*“ OR “agri-food system*“ OR “agrifood chain*“ OR “agri-food chain*“ OR “food industry” OR “food sector” OR “food system” OR “e-commerce” OR “cold chain” OR “wholesale market” OR “market reform*“ OR “market linkage*“ OR “commodity chain*“ OR “commodities chain*“ OR “handicraft*“ OR “artisan*“ OR “self-help group*“ OR “public-private partnership*“ OR “small and medium-size* enterprise*“ OR SMEs OR “small enterprise*“ OR “rural enterprise*“ OR “micro-enterprise*“ OR “microenterprise*“) OR TITLE-ABS-KEY((vertical OR horizontal) W/2 (“integration” OR “coordination” OR “linkage*“)) | 763,607 |
| 3  Value-chain | TITLE-ABS-KEY((“crop” OR “crops” OR “food” OR “produce” OR “agri-product*“ OR “agro-food” OR “tuber*“ OR “root crop*“ OR “barley” OR “oat*“ OR “rye” OR “wheat” OR “arrowroot” OR “artichoke*“ OR “banana*“ OR “yam*“ OR “breadfruit” OR “chickpea*“ OR “lentil*“ OR “pea*“ OR “bean*“ OR “potato*“ OR “cassava*“ OR “millet” OR “rice” OR “amaranth” OR “paddy” OR “maize” OR “sorghum” OR “corn” OR “cashew*“ OR “meat” OR “fish” OR “vegetable*“ OR “chicken” OR “turkey” OR “duck” OR “fruit” OR “staple crop*“ OR “cash crop*“ OR “rubber” OR “plantain*“ OR “sugarcane” OR “timber” OR “cotton” OR “coffee” OR “tea” OR “bean*“ OR “legume*“ OR “spice*“ OR “livestock” OR “pork” OR “poultry” OR “shrimp” OR “cattle” OR “cow*“ OR “beef” OR “pig*“ OR “goat*“ OR “sheep” OR “milk” OR “dairy” OR “tomato*“ OR “carrot*“ OR “onion*“ OR “cauliflower” OR “grain*“ OR “cereal” OR “soybean*“ OR “peanut*“ OR “oilseed*“ OR “citrus” OR “fodder” OR “hay” OR “silage” OR “forage” OR “palm” OR “melon*“ OR “avocado*“ OR “flower*“ OR “pulse*“ OR “ground nut*“ OR “egg*“ OR “strawberr*“ OR “currant*“ OR “*berry” OR “*berries” OR “mango*“ OR “guava*“ OR “papaya*“ OR “pawpaw” OR “paw-paw” OR “orange*“ OR “lemon*“ OR “spinach” OR “lettuce” OR “mushroom*“ OR “pepper*“ OR “microgreen*“) W/3 (“processing” OR “packaging” OR “trade” OR “trading” OR “retail*“ OR “transport*“ OR “distribution” OR “storage” OR “storing” OR “branding” OR organic OR certification)) | 394,316 |
| 4 | #2 OR #3 | 1,127,405 |
| 5  Empowerment | TITLE-ABS-KEY(empower* OR disempower* OR autonomy OR (decision* W/2 (make OR made OR making))) OR TITLE-ABS-KEY(self-determin* OR bargain* OR negotiat* OR equal* OR agency OR transformati* OR particip* OR engag* OR inclus* OR represen* OR access* OR equit* OR inequit* OR inequalit* OR disadvantage* OR marginali* OR discriminat* OR vulnerab* OR “self help” OR ownership* OR power* OR norm OR norms OR poverty OR economic*) | 18,532,952 |
| 6  Women | TITLE-ABS-KEY(gender* or woman* or women* or mother* or maternal or female* or wife* or wives) | 11,866,371 |
| 7  LMIC | TITLE-ABS-KEY(afghanistan or albania or algeria or “american samoa” or angola or “antigua and barbuda” or antigua or barbuda or argentina or armenia or armenian or aruba or azerbaijan or bahrain or bangladesh or barbados or belarus or byelarus or belorussia or byelorussian or belize or “british honduras” or benin or dahomey or bhutan or bolivia or bosnia or herzegovina or botswana or bechuanaland or brazil or brasil or bulgaria or “burkina faso” or “burkina fasso” or “upper volta” or burundi or urundi or “cabo verde” or “cape verde” or cambodia or kampuchea or “khmer republic” or cameroon or cameron or cameroun or “central african republic” or “ubangi shari” or chad or chile or china or colombia or comoros or “comoro islands” or “iles comores” or mayotte or congo or zaire or “costa rica” or “cote d ivoire” or “cote divoire” or “cote d ivoire” or “ivory coast” or croatia or cuba or cyprus or “czech republic” or czechoslovakia or djibouti or “french somaliland” or dominica or “dominican republic” or ecuador or egypt or “united arab republic” or “el salvador” or eritrea or estonia or eswatini or swaziland or ethiopia or fiji or gabon or “gabonese republic” or gambia or “georgia (republic)” or georgian or ghana or “gold coast” or gibraltar or greece or grenada or guam or guatemala or guinea or guyana or “british guiana” or haiti or hispaniola or honduras or hungary or india or indonesia or timor or iran or iraq or “isle of man” or jamaica or jordan or kazakhstan or kazakh or kenya or korea or kosovo or kyrgyzstan or kirghizia or kirgizstan or “kyrgyz republic” or kirghiz or laos or “lao pdr” or “lao people’s democratic republic” or latvia or lebanon or “lebanese republic” or lesotho or basutoland or liberia or libya or “libyan arab jamahiriya” or lithuania or macau or macao or macedonia or madagascar or “malagasy republic” or malawi or nyasaland or malaysia or “malay federation” or “malaya federation” or maldives or “indian ocean” or mali or malta or micronesia or kiribati or “marshall islands” or nauru or “northern mariana islands” or palau or tuvalu or mauritania or mauritius or mexico or moldova or moldovian or mongolia or montenegro or morocco or ifni or mozambique or “portuguese east africa” or myanmar or burma or namibia or nepal or “netherlands antilles” or nicaragua or niger or nigeria or oman or muscat or pakistan or panama or “papua new guinea” or “new guinea” or paraguay or peru or philippines or philipines or phillipines or phillippines or poland or “polish people’s republic” or portugal or “portuguese republic” or “puerto rico” or romania or russia or “russian federation” or ussr or “soviet union” or “union of soviet socialist republics” or rwanda or ruanda or samoa or “pacific islands” or polynesia or “samoan islands” or “navigator island” or “navigator islands” or “sao tome and principe” or “saudi arabia” or senegal or serbia or seychelles or “sierra leone” or slovakia or “slovak republic” or slovenia or melanesia or “solomon island” or “solomon islands” or “norfolk island” or “norfolk islands” or somalia or “south africa” or “south sudan” or “sri lanka” or ceylon or “saint kitts and nevis” or “st. kitts and nevis” or “saint lucia” or “st. lucia” or “saint vincent and the grenadines” or “saint vincent” or “st. vincent” or grenadines or sudan or suriname or surinam or “dutch guiana” or “netherlands guiana” or syria or “syrian arab republic” or tajikistan or tadjikistan or tadzhikistan or tadzhik or tanzania or tanganyika or thailand or siam or “timor leste” or “east timor” or togo or “togolese republic” or tonga or “trinidad and tobago” or trinidad or tobago or tunisia or turkey or turkmenistan or turkmen or uganda or ukraine or uruguay or uzbekistan or uzbek or vanuatu or “new hebrides” or venezuela or vietnam or “viet nam” or “middle east” or “west bank” or gaza or palestine or yemen or yugoslavia or zambia or zimbabwe or “northern rhodesia” or “global south” or “africa south of the sahara” or “sub-saharan africa” or “subsaharan africa” or “africa, central” or “central africa” or “africa, northern” or “north africa” or “northern africa” or magreb or maghrib or sahara or “africa, southern” or “southern africa” or “africa, eastern” or “east africa” or “eastern africa” or “africa, western” or “west africa” or “western africa” or “west indies” or “indian ocean islands” or caribbean or “central america” or “latin america” or “south and central america” or “south america” or “asia, central” or “central asia” or “asia, northern” or “north asia” or “northern asia” or “asia, southeastern” or “southeastern asia” or “south eastern asia” or “southeast asia” or “south east asia” or “asia, western” or “western asia” or “europe, eastern” or “east europe” or “eastern europe”) | 6,163,381 |
| 8  LMIC | TITLE-ABS-KEY(“developing country” or “developing countries” or “developing nation*“ or “developing population*“ or “developing world” or “less developed countr*“ or “less developed nation*“ or “less developed population*“ or “less developed world” or “lesser developed countr*“ or “lesser developed nation*“ or “lesser developed population*“ or “lesser developed world” or “under developed countr*“ or “under developed nation*“ or “under developed population*“ or “under developed world” or “underdeveloped countr*“ or “underdeveloped nation*“ or “underdeveloped population*“ or “underdeveloped world” or “middle income countr*“ or “middle income nation*“ or “middle income population*“ or “low income countr*“ or “low income nation*“ or “low income population*“ or “lower income countr*“ or “lower income nation*“ or “lower income population*“ or “underserved countr*“ or “underserved nation*“ or “underserved population*“ or “underserved world” or “under served countr*“ or “under served nation*“ or “under served population*“ or “under served world” or “deprived countr*“ or “deprived nation*“ or “deprived population*“ or “deprived world” or “poor countr*“ or “poor nation*“ or “poor population*“ or “poor world” or “poorer countr*“ or “poorer nation*“ or “poorer population*“ or “poorer world” or “developing econom*“ or “less developed econom*“ or “lesser developed econom*“ or “under developed econom*“ or “underdeveloped econom*“ or “middle income econom*“ or “low income econom*“ or “lower income econom*“ or “low gdp” or “low gnp” or “low gross domestic” or “low gross national” or “lower gdp” or “lower gnp” or “lower gross domestic” or “lower gross national” or lmic or lmics or “third world” or “lami countr*“ or “transitional countr*“ or “emerging economies” or “emerging nation*“) | 412,090 |
| 9  LMIC | #7 OR #8 | 6,349,458 |
| 10  All combined concepts | #1 AND #4 AND #5 AND #6 AND #9 | 2,662 |

[Enter text here]

## 2 Appendix B List of organisations/Websites

- Consultative Group on International Agricultural Research  CGIAR,
- The International Fund for Agricultural Development (IFAD),
- AgriProFocus,
- Bill & Melinda Gates Foundation (BMGF),
- Donor Committee for Enterprise Development,
- The Food and Agriculture Organisation FAO,
- International Labour Organisation ILO,
- Netherlands Development Organization,
- The United States Agency for International Development  USAID,
- Swiss Agency for Development and Cooperation
- The International Food Policy Research Institute  IFPRI
- World Agroforestry
- International Livestock Research Institute
- 'British Library for Development Studies (BLDS)
- International System for Agricultural Science and Technology (AGRIS)
- IMMANA grant databsse
- 3ie impact evaluation database
- Innovation for Poverty Action (IPA)
- Abdul Latif Jameel Poverty Action Lab (J-PAL)
- The World Bank Independent Evaluation Group
- The USAID Development
- Experience Clearinghouse
- The proceedings of the Agriculture
- Nutrition and Health Academy conference
- The proceedings of the Centre for the Study of African Economies Conference
- The proceedings of the North East Universities Development Consortium Conference
- The World Bank Economic Review [Enter text here]

## 3 Appendix C- Screening tool

Screening tool for Systematic Review 3 (Value Chain Intervention and women’s empowerment)

1. Is the study conducted in Low and Middle Income Countries, as per the latest World Bank Classification?

- Yes, Include and see Question. 2
- No, Exclude on country

2. Does the study target women or other stakeholders such as smallholder farmers, value chain entrepreneurs or employees engaged in agriculture and food systems, particularly in value chain development and market engagement interventions?

- Yes, Include and See Question. 3
- No, Exclude as Exclude on population

3. Does the study evaluate an intervention (policy, programme, project or practice), or a review of evaluations of an intervention aimed at empowering women through value chain development and market engagement interventions?

- Yes, Include and See Question. 4
- No, Exclude as Exclude on intervention

4. Does the study analyse the effect of the intervention on the empowerment of women? This may include measures such as encouraging or enabling women to participate, lead and become members of farmer organisations?

- Yes, Include and See Question. 5
- No, Exclude as Exclude on outcome

5. Does the study have an experimental or non-experimental design with comparison group, instrumental variables and interrupted time series?

- Yes, Include for effectiveness and Stop!
- No, See Question.6

6. Does the study evaluate a programme/policy for the empowerment of women in agricultural value chain and discusses implementation issues?

- Yes, Include for process evaluation
- No, Exclude as Exclude on design

## 4 Appendix – D Coding Tool

CODING TOOL

| *Category* | *Sub Category* |
| --- | --- |
| *Publication Status* | - Ongoing - Completed |
| *Region* | - East Asia & Pacific - Europe & Central Asia - Latin America & Caribbean - Middle East & North Africa - South Asia - Sub Saharan Africa |
| *Country Name* |  |
| *Settings* | - Rural - Urban - Rural and Urban (Both) - Not Clear |
| *Project/ Intervention Name* |  |
| *Year* |  |
| *Funding agency* |  |
| *Implementing agency* |  |
| *Duration of intervention* |  |
| *Unit of delivery* | - Individual-One to one - Group |
| *Gender* | - Male - Female - Non-Binary - All sexes - Not reported |
| *BAME* | - Mainly/exclusively (80%) - Partly - None - Not clear |
| *Study Design* | - Experimental design - Non- Experimental design - Process Evaluation |
| *Study Method* | - Randomized Controlled Trial - Difference in Difference - Instrumental variable estimation - Regression discontinuity design - Statistical Matching (PSM) - Interrupted time series - Fixed effects estimation |
| *Mixed _method* | - Yes - No |
| *Intervention category* | Intervention sub-category |
| *Value chain development* | - Enabling policies and institutional environment - Financial services (including both grants & subsidies; and micro-credit, savings & insurance), - Processing and storage facilities - Horizontal and vertical coordination - Process, product, and chain upgrading - Enterprise development and impact investing - Promoting the production of a new profitable product. - Improving product market quality (fairtrade, organic farming, and quality standards) - Supporting horizontal integration of producers group to access better prices. - Improving processing techniques - contract-farming |
| *Market engagement* | - Inclusive market systems development - Gender-friendly markets (including lighting, washroom facilities, provision for childcare), - Access to markets (through farm-to-market roads, transport facilities, - Market structures |
| *Outcome Domain* | Outcome Sub-domain |
| *Economic Empowerment* | - Decision-making on value chain activities - Decision-making over the use of income, - Increased bargaining power - Leadership positions in groups |
| *Economic benefits* | - Farm productivity - Income - Time use - Assets ownership |
| *Participation* | - Access to information on production/markets - Enhanced social and institutional networks - Increased participation in paid labor opportunities - Access to new markets - Knowledge and skills - Gender roles and norms |
| *Effect sizes calculation* |  |
| *Economic Empowerment* |  |
| *Economic benefits* |  |
| *Participation* |  |
| *Attrition* |  |
| *Differential Attrition* |  |
| *Barriers and Facilitators to the participation* |  |
| *Barriers and Facilitators to Outcome* |  |
| *Design Issues* |  |
| *Implementation Issues* |  |
| *What target populations say* |  |
| *Moderators and Confounders* |  |

| *Critical appraisal tool for primary studies: effectiveness* | | | |
| --- | --- | --- | --- |
| *Item* | Description | Key | Notes |
| *Intervention* | Is the intervention clearly named and described, including all relevant components. See examples below. | High: full and clear description, so that the main components and how they are delivered are clear  Medium: Partial description  Low: Little or no description |  |
| *Evaluation questions* | Are the evaluation questions clearly stated? | High: full and clear description, so that the main components and how they are delivered are clear  Medium: Partial description  Low: Little or no description |  |
| *Study design* | Use the study design coding | High: Experimental  Medium: Non-experimental  Low: Before versus after |  |
| *Outcomes* | Are the outcomes clearly defined? Where appropriate do they use an existing, validated measurement tool?  See examples below. | High: full and clear definition using validated instruments where available (a researcher wishing to use these outcomes would have sufficient information to do so)  Medium: Partial definition. May use validated instruments but without sufficient references to source.  Low: Little or no definition |  |
| *Sample size (power calculation)* | Do the authors report a power calculation as the basis for sample size? | High: Power calculation report and sample size meets necessary sample size  Medium: Power calculation mentioned and sample size meets necessary sample size  Low: No mention of power calculation. |  |
| *Attrition* | Reported for endline and longest follow up.  Calculate overall attrition and differential attrition (see example below). It is often necessary to calculate from table of results. If sample size varies by outcome calculate for highest attrition. | High: Attrition within IES conservative standard  Medium: Attrition within IES liberal standard  Low: Attrition outside IES liberal standard |  |
| *Overall (including questions for all studies)* | The overall score uses the weakest link in the chain principle i.e., is the lowest score on any item | High: High on all items  Medium: No lower than medium on any item  Low: At least one low |  |

Questions for process evaluations (apply to implementation sections) [used for any study coded as having implementation evidence]

|  |  | *High* | *Medium* | *Low* |  | *Low* |
| --- | --- | --- | --- | --- | --- | --- |
| *1* | Is the qualitative methodology described? | Yes |  | No | >> 3 |  |
| *2* | Is the qualitatively methodology appropriate to address the evaluation questions? | Yes | Partially | No |  | Insufficient detail |
| *3* | Is the recruitment or sampling strategy described? | Yes |  | No | >> 5 |  |
| *4* | Is the recruitment or sampling strategy appropriate to address the evaluation questions? | Yes | Partially | No |  | Insufficient detail |
| *5* | Are the researcher’s own position, assumptions and possible biases outlined? | Yes | Partially | No |  |  |
| *6* | Have ethical considerations been sufficiently considered? | Yes | Partially | No |  | Insufficient detail |
| *7* | Is the data analysis approach adequately described? | Yes |  | No | >>9 |  |
| *8* | Is the data analysis sufficiently rigorous? | Yes | Partially | No |  |  |
| *9* | Are the implications or recommendations clearly based in the evidence from the study? | Yes | Partially | No |  |  |
| *10* | Overall (including questions for all studies- The overall score uses the weakest link in the chain principle i.e., is the lowest score on any item | High: High on all items  Medium: No lower than medium on any item  Low: At least one low |  |  |  |  |

## 5 Appendix E- Definition of the Intervention and Outcome

|  | Categories | Sub- Categories | Definition |
| --- | --- | --- | --- |
|  | Economic Empowerment | Decision-making on value chain activities | Women’s decision-making power (e.g., over agricultural production, income, or household food consumption; reduction of outcomes associated with disempowerment), (e.g. gender based violence, time burden) |
|  |  | Decision-making over the use of income |  |
|  |  | Increased bargaining power | Women’s bargaining power in negotiating and managing vertical and horizontal relationships |
|  |  | Leadership positions in groups | Membership in economic or social groups and ease with speaking in public |
|  |  | Mobility | Increase in women’s mobility in spaces considered to male. Spaces to be gender neutral where information is exchanged.  Also, the environment that restricts women’s mobility reduces their ability to engage in networking opportunities and limits the information available to them. |
|  | Economic benefits | Farm productivity | Increase in farm productivity |
|  |  | Income | Increase in income of the household/individual |
|  |  | Assets ownership | Ownership of, access to, and decision-making power over productive resources such as land, livestock, agricultural equipment, consumer durables, and credit |
|  | Participation | Access to information on production/markets | Access to information about new markets and, production processes |
|  |  | Enhanced social and institutional networks | Increased ability to develop social capital and challenge social constraints  Reduced social pressure for women to conform to stereotypes.  Reduced violence in the household; reduced pressure to conform to social norms.  Financial institution |
|  |  | Increased participation in paid labor opportunities/ Labor participation | Women are considered for all skilled position in production processes as well as in firms engaged in exporting and marketing. |
|  |  | Access to new markets | Access to new markets by Women/Men |
|  |  | Knowledge and skills | Increase in knowledge and skills |
|  |  | Gender roles and norms | Influence gender roles and norms; change in gender roles and norms, |
|  | Time use | Workload | Allocation of time to productive and domestic tasks |
|  |  | Leisure | Satisfaction with the available time for leisure activities |
| **Intervention category** | | **Intervention sub-category** | **Definition** |
| Value chain development | | Enabling policies and institutional environment | Policies that facilitate value chain (VC) development; and institutions that ensure a conducive environment for VC policy implementation |
|  |  | Financial services (including both grants and subsidies, and micro-credit, savings and insurance), | Activities that ensure solutions to monetary challenges that hinder VC development. (e.g., provision of grants, subsidies, investment advice, loans, micro-credit). |
|  |  | Processing and storage facilities | Processing:  Infrastructure that allows preparation/treatment of raw agricultural products into intermediary or finished products. (e.g., ranges from factories to simple household product processing mechanism)  Storage: infrastructure/technology that allows preservation of agricultural products over time (e.g., warehouses, barns, hermetic bags, cold chains). |
|  |  | Horizontal and vertical coordination | Horizontal coordination:  Collaboration by at least two VC actors to jointly perform an activity.  Farmers at one particular link in the value chain may aggregate and coordinate activities through farmers’ based organisations (FBOs) or cooperatives. There are many potential benefits to coordination, (e.g., increase in bargaining power in negotiating prices, exploitation of scale economies, through for example the collective purchase of machinery, risk pooling, and other reductions in transaction costs).  Vertical coordination:  Involves a superior/more efficient VC actor taking over the activities of less efficient actor(s)  This refers to the establishment links between market agents along the vertical chain running from production to final consumption. New links can be established between farmers and buyers that can offer more remunerative prices (contract production).(e.g., farmers may sign profitable supply contracts with supermarket chains). |
|  |  | Process, product, and chain upgrading | Process upgrading: Improving/increasing the efficiency of production (e.g., via use of improved technology, better handling of VC processes etc.)  Product upgrading:  Substituting/replacing a product with an improved version (includes, adding value to an existing product to enhance its quality)  Chain upgrading:  Using/leveraging technology and other linkages to improve the activities of actors along the VC. Chain upgrade also allows actors to improve their efficiency or competitiveness to move into value added activities |
|  |  | Enterprise development and impact investing | Investment of time and capital (both human and material) toward improving the business of a VC actor. It entails a variety of investment strategies that helps VC actors to generate profit and attain specific goals |
|  |  | Promoting the production of a new profitable product. | Entails the identification and development of strategies to boost the production or manufacturing of a novel and profitable product |
|  |  | Improving product market quality (fairtrade, organic farming, and quality standards) | Acquiring and/or participating in established quality standards (e.g., domestic or international) that enhance marketability of products along the VC |
|  |  | Supporting the horizontal integration of producers group to access better prices. | Coordinating the activities of producers of homogeneous or similar products to enhance their bargaining power for better prices |
|  |  | Improving processing techniques | Using more efficient approaches to process products along the VC |
|  |  | contract-farming | Agreement between producers and buyers that states the conditions for production and sale of agricultural products |
| Market engagement | | Inclusive market systems development | Allow participation of the marginalised in market engagement (no discrimination against ‘minority’ groups) |
|  |  | Gender-friendly markets (including lighting, washroom facilities, provision for childcare), | Markets with facilities that address the needs of all participants (especially those of women participants). (E.g., toilet facilities, appropriate waste disposal bins, provision of secured childcare facilities). |
|  |  | Access to markets (through farm-to-market roads, transport facilities, | Ease of entry into market centres: (e.g., motorable roads connecting farms to markets). |
|  |  | Market structures | How firms within a market are classified given the type of competition for their goods and services (e.g., monopoly, oligopoly, perfect competition, monopolistic competition) |
